# Supplementary material for: Composition of Dietary Fatty Acids and Health Risks in Japanese Youths
Source: Nutrients. 2021 Jan 28;13(2):426. doi: 10.3390/nu13020426 (PMC7911182; doi:10.3390/nu13020426)
Supplement: Supplementary file 1 [file nutrients-13-00426-s001.zip › SupplementaryFigS1FA-RiskNutrients20210124.docx]

Supplementary Materials: Figure S1. Subject selection


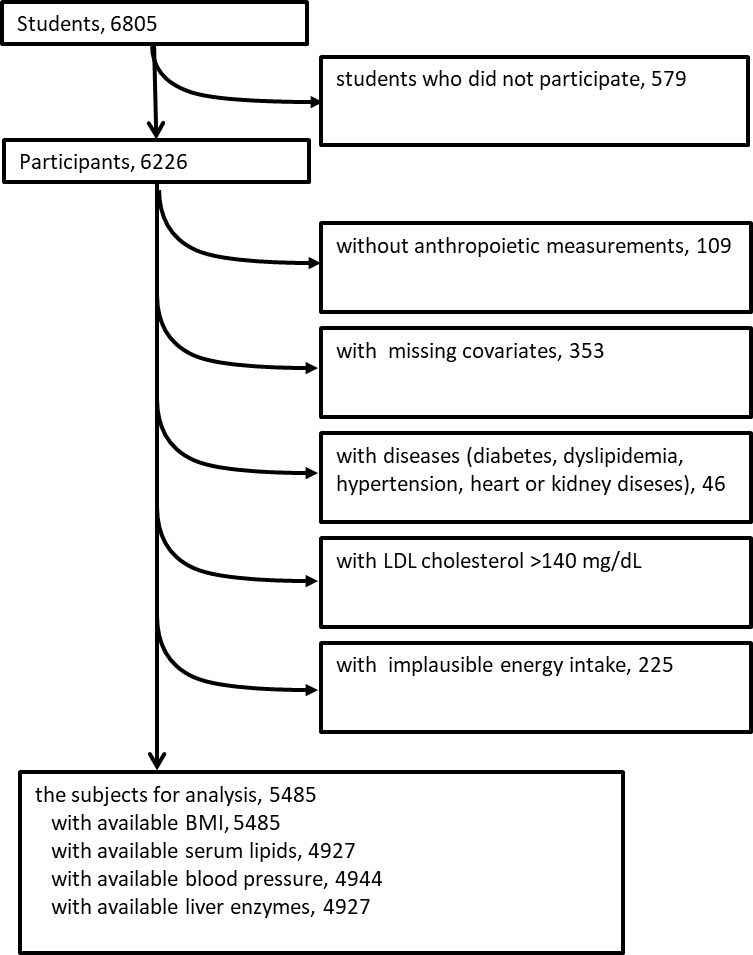


Supplementary Figure S1. Subject selection
