# Supplementary material for: Composition of Dietary Fatty Acids and Health Risks in Japanese Youths
Source: Nutrients. 2021 Jan 28;13(2):426. doi: 10.3390/nu13020426 (PMC7911182; doi:10.3390/nu13020426)
Supplement: Supplementary file 1 [file nutrients-13-00426-s001.zip › SupplementaryFigS2FA-RiskNutrients20210123.docx]

Supplementary Materials: Figure S2. Coefficients of compositional regression models including combinations of energy, protein, fat, and carbohydrates as confounders

**Supplementary Figure S2.** Coefficients of fatty acids for cardiometabolic risk factors in the linear regression models including combinations of energy (E; kcal), protein (P; %E), fat (F) ; %E, and carbohydrate (C; %E), additionally adjusted for age, sex, zBMI, sodium, physical activity, sleeping duration, screen time, single parent, and number of siblings. The linear regression models for zBMI did not include zBMI as a confounder. Bar plots are depicted for the models that showed significant coefficients in **Table 2**.
