# Supplementary material for: Composition of Dietary Fatty Acids and Health Risks in Japanese Youths
Source: Nutrients. 2021 Jan 28;13(2):426. doi: 10.3390/nu13020426 (PMC7911182; doi:10.3390/nu13020426)
Supplement: Supplementary file 1 [file nutrients-13-00426-s001.zip › SupplementaryTableS1FA-RiskNutrients20210124.docx]

Supplementary Materials: Table S1.

**Table S1.** Compositional regression models adjusted for confounders including fat

|  | SFA |  | MUFA |  | omega-6 PUFAs | | omega-3 PUFAs | |
| --- | --- | --- | --- | --- | --- | --- | --- | --- |
|  | *β* (SE) | *p* | *β* (SE) | *p* | *β* (SE) | *p* | *β* (SE) | *p* |
| Height, cm | -0.29 (0.76) | 0.705 | 0.78 (1.68) | 0.642 | -0.14 (1.05) | 0.896 | -0.35 (0.65) | 0.587 |
| Weight, kg | -1.09 (0.98) | 0.263 | 2.55 (2.15) | 0.234 | -1.60 (1.34) | 0.232 | 0.14 (0.83) | 0.862 |
| zBMI | -0.01 (0.11) | 0.906 | 0.10 (0.24) | 0.664 | -0.19 (0.15) | 0.193 | 0.1 (0.09) | 0.265 |
| log(LDL-C, mg/dL) | 0.02 (0.03) | 0.454 | 0.08 (0.06) | 0.161 | **-0.11 (0.04)** | **0.004** | 0.00 (0.02) | 0.905 |
| log(HDL-C, mg/dL) | 0.03 (0.02) | 0.184 | -0.03 (0.05) | 0.581 | 0.01 (0.03) | 0.855 | -0.01 (0.02) | 0.669 |
| SBP, mmHg | -0.81 (1.34) | 0.548 | 1.44 (2.95) | 0.625 | 1.77 (1.83) | 0.334 | **-2.40 (1.15)** | **0.037** |
| DBP, mmHg | -0.35 (1.04) | 0.733 | 0.57 (2.28) | 0.804 | 2.00 (1.42) | 0.159 | **-2.21 (0.89)** | **0.013** |
| log(AST, IU/L) | 0.01 (0.03) | 0.723 | -0.01 (0.06) | 0.900 | -0.02 (0.04) | 0.546 | 0.02 (0.02) | 0.385 |
| log(ALT, IU/L) | 0.04 (0.04) | 0.291 | -0.10 (0.09) | 0.266 | -0.03 (0.06) | 0.570 | **0.09 (0.04)** | **0.012** |
| log(GGT, IU/L) | **0.07 (0.03)** | **0.022** | -0.08 (0.07) | 0.235 | -0.02 (0.04) | 0.544 | 0.03 (0.03) | 0.187 |

SFA, MUFA, and PUFA: saturated, monounsaturated, polyunsaturated fatty acids; SE: standard error; zBMI: z score of body mass index; LDL-C and HDL-C: low- and high-density-lipoprotein cholesterol; SBP and DBP: systolic and diastolic blood pressure; AST and ALT: aspartate and alanine transaminase; GGT: gamma glutamyl transpeptidase. Regression coefficients for the first part of the compositional fatty acids were obtained. Coefficients (*β*) for height, weight, and zBMI were in the linear regression models with the following confounders: age, sex, energy (kcal), fat (%E), sodium, physical activity, sleeping duration, screen time, single parent, and number of siblings. Coefficients for other risks were in the linear regression models with the same confounders plus zBMI. Coefficients for risk levels correspond to an increase in the consumption of each fatty acid relative to other fatty acids.
