# Supplementary material for: Composition of Dietary Fatty Acids and Health Risks in Japanese Youths
Source: Nutrients. 2021 Jan 28;13(2):426. doi: 10.3390/nu13020426 (PMC7911182; doi:10.3390/nu13020426)
Supplement: Supplementary file 1 [file nutrients-13-00426-s001.zip › SupplementaryTableS2FA-RiskNutrients20210124.docx]

Supplementary Materials: Table S2

**Table S2**. Coefficients of linear regression models with a composition of all energy-generating nutrients.

|  | SFA |  |  | MUFA |  |  | omega-6 PUFAs | |  | omega-3 PUFAs | |  |
| --- | --- | --- | --- | --- | --- | --- | --- | --- | --- | --- | --- | --- |
|  | *β* (SE) | *p* |  | *β* (SE) | *p* |  | *β* (SE) | *p* |  | *β* (SE) | *p* |  |
| Height, cm | -0.78 (0.81) | 0.333 |  | 0.27 (1.56) | 0.862 |  | -0.32 (1.02) | 0.751 |  | -0.43 (0.69) | 0.529 |  |
| Weight, kg | -2.36 (1.03) | 0.022 | * | 2.64 (2.00) | 0.187 |  | -1.64 (1.30) | 0.208 |  | -0.70 (0.88) | 0.427 |  |
| zBMI | -0.15 (0.11) | 0.176 |  | 0.15 (0.22) | 0.507 |  | -0.18 (0.14) | 0.219 |  | -0.01 (0.10) | 0.885 |  |
| Log(LDL-C, mg/L) | 0.04 (0.03) | 0.144 |  | 0.08 (0.06) | 0.140 |  | -0.11 (0.04) | 0.003 | ** | 0.02 (0.02) | 0.351 |  |
| Log(HDL-C mg/L) | 0.03 (0.03) | 0.293 |  | -0.02 (0.05) | 0.676 |  | 0.02 (0.03) | 0.489 |  | -0.03 (0.02) | 0.248 |  |
| SBP, mmHg | 0.43 (1.42) | 0.764 |  | 1.27 (2.75) | 0.644 |  | 0.87 (1.78) | 0.626 |  | -0.77 (1.21) | 0.527 |  |
| DBP, mmHg | 1.09 (1.10) | 0.321 |  | 0.76 (2.13) | 0.722 |  | 1.38 (1.38) | 0.316 |  | -0.70 (0.94) | 0.455 |  |
| Log(AST, IU/L) | -0.03 (0.03) | 0.334 |  | -0.01 (0.05) | 0.783 |  | -0.01 (0.03) | 0.814 |  | -0.01 (0.02) | 0.545 |  |
| Log(ALT, IU/L) | -0.02 (0.04) | 0.628 |  | -0.10 (0.08) | 0.224 |  | 0.00 (0.05) | 0.951 |  | 0.02 (0.04) | 0.552 |  |
| Log(GGT, IU/L) | 0.04 (0.03) | 0.248 |  | -0.07 (0.06) | 0.237 |  | -0.01 (0.04) | 0.852 |  | 0.00 (0.03) | 0.969 |  |

SFA, MUFA,, and PUFA: saturated, monounsaturated, and polyunsaturated fatty acids; β: coefficient of linear regression model; SE, standard error; zBMI: z score of body mass index; LDL-C and HDL-C: low- and high-density-lipoprotein cholesterol; SBP and DBP: *β*systolic and diastolic blood pressure; AST and ALT: aspartate and alanine transaminase; GGT: gamma glutamyl transpeptidase. Regression coefficients for the first part of the compositional energy-generating nutrients were obtained. Energy-generating nutrients were SFA, MUFA, omega-6 PUFAs, omega-3 PUFAs, protein, carbohydrate, and non-FA fat. Coefficients indicate an increase in each fatty acid relative to other fatty acids. When height, weight, and zBMI were dependent variables, the confounders were age, sex, energy (kcal), sodium, physical activity, sleeping duration, screen time, single parent, and number of siblings. When TG, LDL-C and HDL-C, systolic and diastolic blood pressure, AST, ALT, and GGT were dependent variables, the same confounders plus zBMI were used. *: *p* < 0.05; **: *p* < 0.01.

Continued Table S2.

|  | Protein |  |  | Carbohydarate | |  | non-FA fat | |  |
| --- | --- | --- | --- | --- | --- | --- | --- | --- | --- |
|  | *β* (SE) | *p* |  | *β* (SE) | *p* |  | *β* (SE) | *p* |  |
| Height, cm | 0.12 (0.78) | 0.878 |  | 0.95 (0.62) | 0.129 |  | 0.27 (0.61) | 0.657 |  |
| Weight, kg | 3.41 (0.99) | 0.001 | *** | 1.12 (0.8) | 0.162 |  | -2.30 (0.78) | 0.003 | ** |
| zBMI | 0.42 (0.11) | <0.001 | *** | 0.02 (0.09) | 0.782 |  | -0.26 (0.09) | 0.003 | ** |
| Log(LDL-C, mg/L) | -0.04 (0.03) | 0.152 |  | 0.03 (0.02) | 0.134 |  | -0.01 (0.02) | 0.543 |  |
| Log(HDL-C mg/L) | 0.02 (0.02) | 0.393 |  | -0.06 (0.02) | 0.004 | ** | 0.03 (0.02) | 0.115 |  |
| SBP, mmHg | -2.95 (1.37) | 0.031 | * | 2.43 (1.09) | 0.026 | * | -0.99 (1.09) | 0.363 |  |
| DBP, mmHg | -2.65 (1.06) | 0.013 | * | 1.30 (0.85) | 0.125 |  | -0.99 (0.84) | 0.241 |  |
| Log(AST, IU/L) | 0.06 (0.03) | 0.019 | * | -0.03 (0.02) | 0.157 |  | 0.03 (0.02) | 0.209 |  |
| Log(ALT, IU/L) | 0.13 (0.04) | 0.002 | ** | -0.08 (0.03) | 0.021 | * | 0.03 (0.03) | 0.312 |  |
| Log(GGT, IU/L) | 0.07 (0.03) | 0.030 | * | -0.06 (0.02) | 0.014 | * | 0.02 (0.02) | 0.338 |  |
