# Supplementary material for: Composition of Dietary Fatty Acids and Health Risks in Japanese Youths
Source: Nutrients. 2021 Jan 28;13(2):426. doi: 10.3390/nu13020426 (PMC7911182; doi:10.3390/nu13020426)
Supplement: Supplementary file 1 [file nutrients-13-00426-s001.zip › SupplementaryTableS3OverweightNutrients20210125.docx]

Supplementary Materials: Table S3.

**Table S3. The proportion of the subjects with BMI >1.0 in each quintile category of cardiometabolic risks (%).**

|  | Q1 | Q2 | Q3 | Q4 | Q5 |
| --- | --- | --- | --- | --- | --- |
| **Height** | 6.4 | 8.4 | 9.2 | 9.2 | 12.2 |
| **Weight** | 0.0 | 0.1 | 0.2 | 3.0 | 42.1 |
| **Body mass index** | 0.0 | 0.0 | 0.0 | 0.0 | 45.4 |
| **Low density lipoprotein cholesterol** | 5.9 | 6.9 | 9.6 | 8.2 | 12.5 |
| **High density lipoprotein cholesterol** | 17.8 | 10.9 | 6.7 | 5.1 | 2.6 |
| **Systolic blood pressure** | 2.9 | 6.1 | 8.8 | 10.8 | 15.2 |
| **Diastolic blood pressure** | 3.4 | 5.8 | 7.9 | 10.2 | 16.5 |
| **Aspartate transaminase** | 15.2 | 9.0 | 6.7 | 5.5 | 6.7 |
| **Alanine transaminase** | 7.9 | 7.5 | 5.7 | 6.5 | 15.5 |
| **Gamma glutamyl transpeptidase** | 8.1 | 6.3 | 8.0 | 7.4 | 13.3 |
